# *Aplasia cutis congenita* as a complication of early interstitial laser embryo reduction in a case of monochorionic triamniotic triplet pregnancy ‒ a case report

**DOI:** 10.1515/crpm-2020-0078

**Published:** 2024-05-07

**Authors:** Anita Kaul, Chanchal Singh, Ila Gupta

**Affiliations:** Apollo Centre for Fetal Medicine, New Delhi, India; Max Hospital, New Delhi, India

**Keywords:** interstitial laser, fetal reduction, *Aplasia cutis congenita*

## Abstract

**Objectives:**

Monochorionic multiple pregnancies are being increasingly encountered. Early embryo reduction by interstitial laser is an option of therapeutic intervention. The patient counseling is mainly focused on miscarriage and fetal demise but this case report highlights that the counseling also needs to mention the possibility of developing *Aplasia cutis congenita* post intervention and that interstitial laser though technically feasible can have rare complications.

**Case presentation:**

This was a uncommon form of twinning diagnosed as monochorionic triamniotic triplet pregnancy where the parents wanted to continue with a single fetus. Interstitial laser was performed in two of the embryos, which became papyraceous. The newborn on delivery was seen to have extensive skin scarring on the trunk which was diagnosed as *Aplasia cutis congenita*.

**Conclusions:**

Awareness on complications on early interstitial laser procedures beyond miscarriage and fetal demise and the psychological impact on the parents of seeing the scarred neonate for the first time, if they have not been made aware of this complication.

## Introduction

Monochorionic multiple pregnancies are increasingly recognized on early scans and offered interstitial laser as a means of selective reduction. The easy availability of laser and other minimal invasive technique has made this method of reduction a popular choice amongst many specialists in India. This case illustrates that caution still needs to be exercised before offering reduction in the late first trimester or early second trimester of pregnancy, as at this gestation the reduced fetus becomes papyraceous rather than getting resorbed. The chances of cutaneous infarcts is higher in this situation than cerebral infarcts thus potentiating *Aplasia cutis congenita* (ACC).

## Case presentation

A 34-year old primigravida was seen following a spontaneous conception after subfertility treatment at 12 weeks and 2 days of pregnancy. She had been diagnosed as a monochorionic tri-amniotic pregnancy elsewhere, which was reconfirmed and was counseled about the risks of continuing as a triplet pregnancy/monochorionic complications. The couple were clear that they would continue only as a singleton pregnancy to minimize the risks of preterm labor. There were no relevant medical, family or genetic history.

### Clinical findings

The crown-rump length (CRL) of the three fetuses were 64, 71, 74 mm respectively. The adjusted risk for Trisomy 21 based on ultrasound markers (nuchal translucency, nasal bone, ductus venosus Doppler, tricuspid regurgitation) was low risk all three with risk of 1:7,726.

On tracing the umbilical cord insertions, it was decided that the fetus with the most central cord insertion would not be reduced and the two peripheral twins with the marginal insertions will be reduced. They were consented for risks of miscarriage and neurological outcome.

### Procedure

The single placenta was located on the anterior uterine wall. The sac of fetus 3 was accessed laterally and distended with warm saline first through a 22 G spinal needle to ensure there was enough distance between fetus 3 and fetus 2. Thereafter, a 18 G Cook needle was inserted into the fetus just behind the umbilical cord insertion. A 600 μm laser fiber was then introduced in so that 5 mm was advanced beyond the needle tip. Three bursts of 20 W lasting 30 s was applied through the biolitic diode laser until cessation of blood was documented in the umbilical cord and there was hyperechogenicity of the tissues.

Fetus 1 was accessed transplacentally and the similar reduction technique was followed as like fetus 3. We noted slight intra-amniotic bleeding following transplacental access into the sac of fetus 1.

At the end of the procedure, the surviving fetus 2 did not show any changes in middle cerebral artery (MCA) ductus venosus (DV) blood flow pattern.

The procedure time (insertion of 22 G needle to removal of laser fiber) was 1 h 50 min.

Progesterone support (injection 17-hydroxyprogesterone caproate 500 mg intramuscular) was administered postoperatively for prophylactic uterine quiescence along with a single dose of intravenous cefuroxime as per unit protocol.

### Follow-up and outcome

The patient was followed up regularly by serial scans. There was increased alpha-fetoprotein (AFP) concentration in the second trimester. As the anomaly scan was normal she was reassured that this finding is present in over 90 % pf post reduction pregnancies. There was onset of late fetal growth restriction with growth along 9th centile for gestation and delivery was undertaken at 37 weeks and 2 days due to increased resistance in the fetal umbilical arteries.

**Figure 1:**
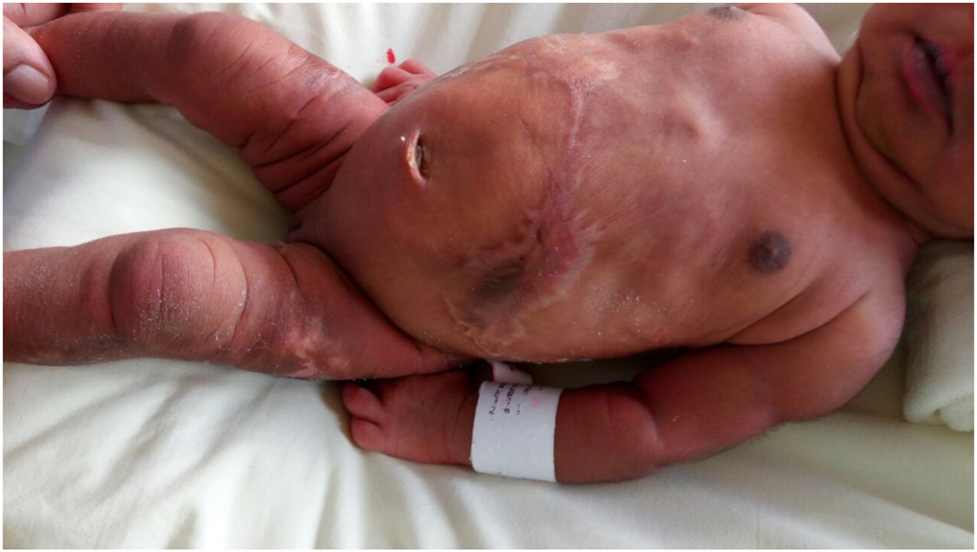
Skin lesions suggestive of *Aplasia cutis congenita* on the anterior aspect of the thorax, abdomen and thighs.

**Figure 2:**
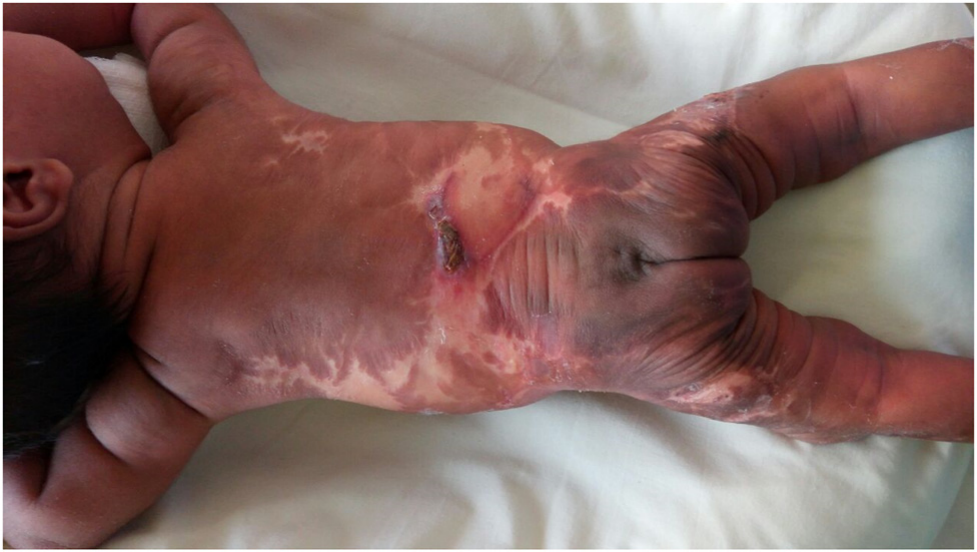
Stellate skin lesions on the back of the neonate.

A female baby with birth-weight of 2,600 g and APGAR score of 9.9 at 1 and 5 min was delivered. There were extensive scars both on the anterior abdominal wall and the back (Figures 1 and 2). This appearance was suggestive of ACC.

**Figure 3:**
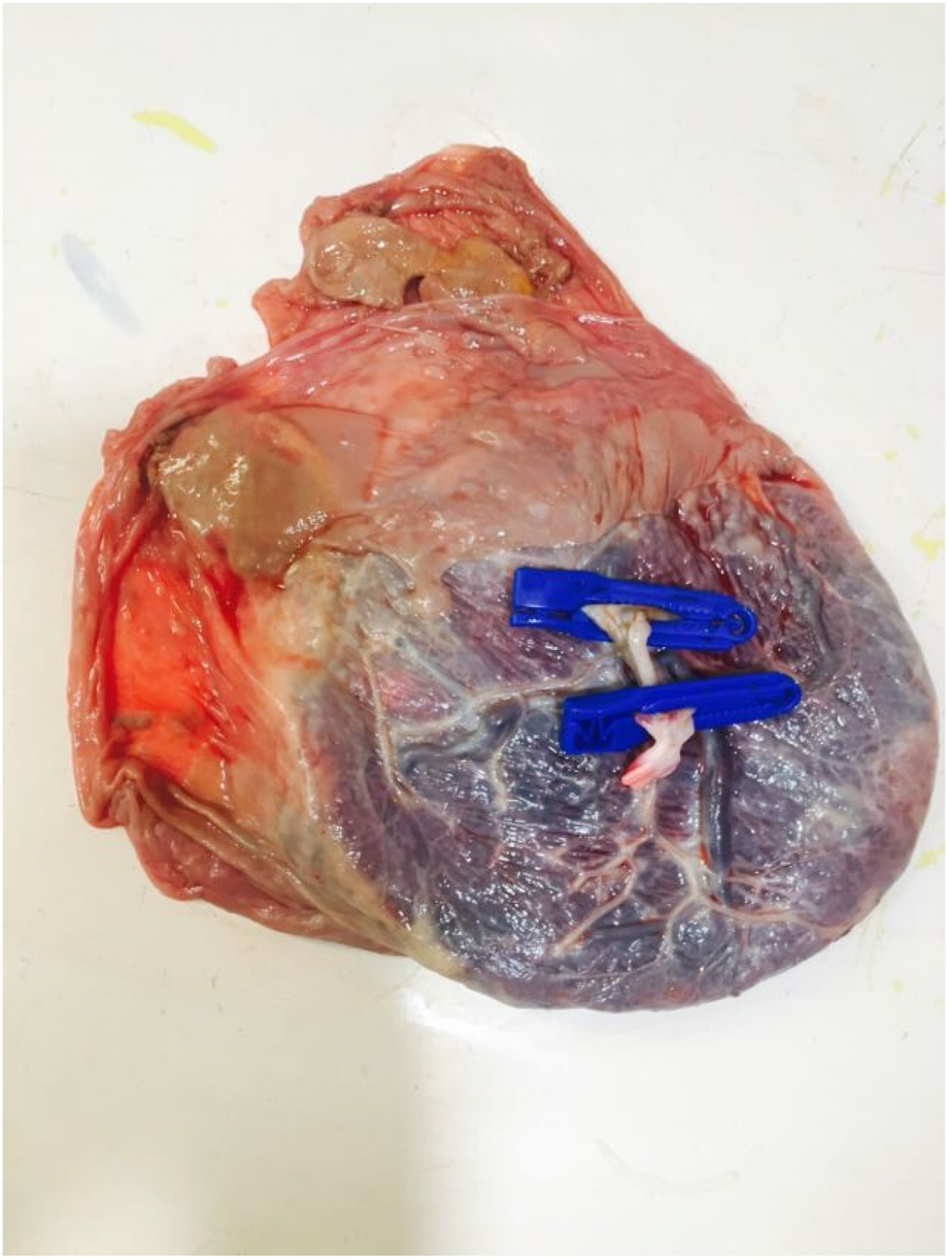
Placenta with two fetus papyraceous.

**Figure 4:**
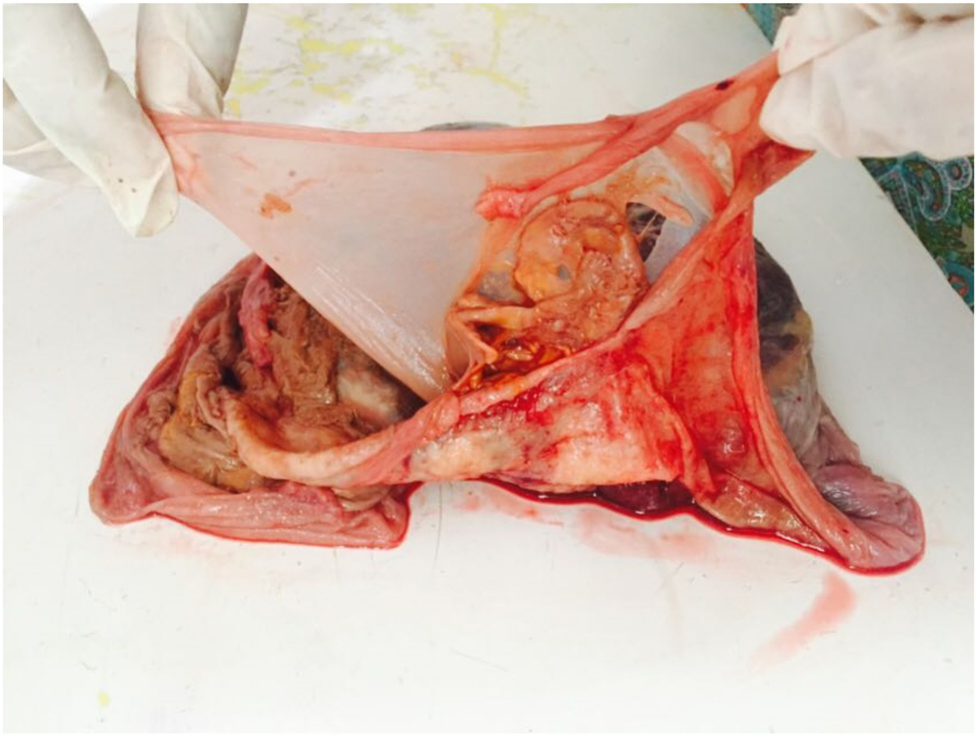
Fetus papyraceous.

Examination of the placenta post-delivery further confirmed the chorionicity and showed the presence of two feti papyraceous (Figures 3 and 4).

## Discussion

ACC is a rare condition defined as absent or scarred areas of skin at birth. It is characterized by the complete absence of all layers of skin in the affected area. Widespread, symmetric, stellate lesions affecting the trunk and extremities have typically been described in association with ‘fetus papyraceous’ in monozygotic pregnancies [1]. The proposed mechanism is that the sudden hypotension associated with one fetal demise in monochorionic pregnancy leads to skin ischemia (feto-fetal transfusion) [2]. Other proposed mechanisms include DIC and emboli from the fetus papyraceous, amniotic membrane adherence; abnormal elastic fiber biomechanical forces; and teratogenic medications such as marijauna and cocaine the exact theory is not known. Infrequently, direct laser burns have been described as a causative factor for ACC, however in such cases, the lesions tend to be asymmetrical, whereas in our case, the lesions were symmetrically present over the anterior abdominal wall and back, making the association unlikely [3].

AFP is a marker of tissue damage or exposed skin, so unusually high levels may be associated with ACC [4]. To the best of our knowledge, only a few cases have been reported following intra fetal laser in complicated monochorionic pregnancies. A series reported by Donoghue et al. supported the association between ACC and early fetal death of a co-twin in a monochorionic pregnancy. They reported ACC in 2 out of 26 neonates (8 %) who had undergone interstitial laser. Most cases of ACC will gradually heal with conservative management with a median time interval of 4 weeks.

Although more recent series with larger cohorts have not reported any case of ACC following intra fetal laser [5, 6], we feel that this case should be reported to highlight that rare complications like ACC can still occur after a successful procedure.

## Conclusions

Although intra fetal laser is a technically feasible procedure for first trimester multifetal pregnancy reduction in monochorionic pregnancies, and rapidly gaining ground in India, clinicians should be aware of the rare complication of ACC that can be visually distressing for parents. Unusually increased AFP levels in the second trimester may be a marker to the underlying presence of ACC.

## Patient perspective

“It was a shock for us when we first saw the baby as we thought that these were laser burns until we met the dermatologist who reassured us that this was a known skin lesion which would lessen over time, which indeed it has.”
